# Response to Misoprostol Treatment in Early Pregnancy Loss: A Single-Center Prospective Observational Study

**DOI:** 10.3390/jcm14238275

**Published:** 2025-11-21

**Authors:** Silvia D’Ippolito, Tina Pasciuto, Chiara Granieri, Sara Giuliano, Greta Barbaro, Giandomenico Pianese, Carmelinda Martino, Chiara Tersigni, Alessio Colalillo, Ursula Catena, Pantaleo Greco, Giuseppe Vizzielli, Tullio Ghi, Francesco Cosentino, Nicoletta Di Simone

**Affiliations:** 1 Dipartimento di Medicina e di Scienze della Salute “Vincenzo Tiberio” (DiMeS), Università degli Studi del Molise, Via Francesco De Sanctis, 86100 Campobasso, Italy; 2 Dipartimento di Scienze della Salute della Donna, del Bambino e di Sanità Pubblica, Fondazione Policlinico Universitario A. Gemelli Istituto di Ricovero e Cura a Carattere Scientifico (I.R.C.C.S.), Largo A. Gemelli 8, 00168 Rome, Italy; 3 Research Core Facility Data Collection G-STeP, Fondazione Policlinico Universitario A. Gemelli Istituto di Ricovero e Cura a Carattere Scientifico (I.R.C.C.S.), Largo A. Gemelli 8, 00168 Rome, Italy; 4 Section of Hygiene, University Department of Life Sciences and Public Health, Università Cattolica Del Sacro Cuore, 00168 Rome, Italy; 5 Faculty of Medicine and Surgery, Università Cattolica Del Sacro Cuore, 00168 Rome, Italysaragiuliano.obgyn@gmail.com (S.G.);; 6 Department of Medical Sciences, Institute of Obstetrics and Gynecology, University of Ferrara, 44122 Ferrara, Italy; 7 Department of Medicine, University of Udine, 33100 Udine, Italy; 8 Clinic of Obstetrics and Gynecology, “Santa Maria della Misericordia” University Hospital, Azienda Sanitaria Universitaria Friuli Centrale, 33100 Udine, Italy; 9 Dipartimento di Scienze della Vita e Sanità Pubblica, Università Cattolica del Sacro Cuore, Largo F. Vito 1, 00168 Rome, Italy; 10 Department of Biomedical Sciences, Humanitas University, Via Rita Levi Montalcini 4, Pieve Emanuele, 20072 Milan, Italy; 11 IRCCS Humanitas Research Hospital, Via Manzoni 56, Rozzano, 20089 Milan, Italy

**Keywords:** spontaneous abortion, dilatation and curettage, misoprostol, prostaglandin

## Abstract

**Introduction:** Medical treatment with misoprostol represents one of the main treatments for Early Pregnancy Loss (EPL). In our study, we aimed to identify clinical features associated with a successful response to this approach. **Methods:** A prospective single-center observational study was conducted at the EPL Clinic of the Department of Obstetrics and Gynecology, Fondazione A. Gemelli IRCCS Rome, Italy. Patients were categorized according to the type of treatment received: spontaneous delivery, elective dilatation and curettage, or medical treatment. A separate analysis was performed within the medical treatment group to distinguish women with a successful response from those with an unsuccessful one. The success of medical treatment was calculated as the response rate (number of patients who successfully responded to treatment/total number of treated patients) with a 95% Confidence Interval (CI). For patients undergoing misoprostol treatment, a multivariable analysis was planned to identify predictors of a successful response, including variables with a *p* value less than 0.05 in the univariable analysis. Receiver Operating Characteristic (ROC) analysis was performed to evaluate the predictive ability of continuous obstetrics parameters for medical treatment success. The optimal cut-off value to differentiate responsive from unresponsive patients was also determined. The significance level was set at *p* < 0.05. **Results:** Sixty-four patients who underwent medical treatment were analyzed. Amenorrhea age was the only parameter inversely associated with treatment success, indicating that an earlier amenorrhea age correlated with a better response to misoprostol. ROC analysis identified a cut-off of 62.5 days, with an AUC (95% CI) of 0.72 (0.55–0.89). An amenorrhea age of ≤ 62.5 days predicted a successful response to medical treatment with a specificity (95% CI) of 90.0% (89.8–90.2) and sensitivity (95% CI) of 54.9% (41.2–68.6). **Conclusions:** Amenorrhea age emerged as a potential predictor of treatment response in women with EPL undergoing misoprostol therapy. However, further studies of larger sample sizes are needed to validate and improve our model.

## 1. Introduction

Early pregnancy loss (EPL) is defined as an intrauterine pregnancy with either an empty gestational sac or a gestational sac containing an embryo/fetus without fetal heart activity within the first 12 weeks of gestation [1]. It is a common pregnancy complication, occurring in about 15–20% of clinically recognized pregnancies and accounting for approximately 80% of all cases of pregnancy loss [1,2,3,4]. Management options for EPL include expectant management, surgical intervention (e.g., Dilation and Curettage, D&C, or suction aspiration), and medical treatment with prostaglandin (PG) analogues with or without anti-progesterone drugs.

Expectant management allows for the spontaneous passage of missed miscarriage. The success rate of this approach ranges from 75% to 95% after one month while the main limitations are related to the unpredictability of the time required for EPL resolution, which may take from 2 to 4 weeks after diagnosis [5,6,7,8,9]. Furthermore, concerns about pain, bleeding, and the potential need for subsequent surgical evacuation may influence the decision process regarding EPL management [7].

Surgical treatment, including D&C or suction aspiration with or without medical cervical preparation, represents a fast, effective, and safe procedure for the removal of spontaneously miscarried tissue from the uterus. However reported complications include bleeding, infection, retained placental or fetal tissue, intrauterine adhesions and, rarely, uterine perforation or cervical trauma, with an overall complication rate of approximately 6% [8,9,10,11].

Medical management of EPL is based on the administration of prostaglandin (PG) analogue misoprostol alone or in combination with the anti-progesterone drug mifepristone [12,13,14]. These drugs are able to ripen the cervix, induce uterine contractility and facilitate the expulsion of miscarried tissue. The most used standard regimen consists of vaginal administration of 800 mcg of misoprostol, with excellent safety and toxicity profiles. In women diagnosed with EPL, in the absence of active bleeding, the complete expulsion rate after standard misoprostol administration varies significantly, from 25% to 86%. About 15–40% of women require a second dose, or ultimately need for a surgical uterine evacuation to expedite treatment [10,11,15,16,17,18].

A recent Cochrane assessing the effectiveness of methods for miscarriage management found that surgical and medical treatments may be more effective than expectant management for achieving a complete uterine evacuation [19]. In particular, suction aspiration after cervical preparation (risk ratio (RR) 2.12, 95% confidence interval (CI) 1.41 to 3.20, low-certainty evidence); dilatation and curettage (RR 1.49, 95% CI 1.26 to 1.75, low-certainty evidence); suction aspiration (RR 1.44, 95% CI 1.29 to 1.62, low-certainty evidence); mifepristone plus misoprostol (RR 1.42, 95% CI 1.22 to 1.66, moderate-certainty evidence); and misoprostol (RR 1.30, 95% CI 1.16 to 1.46, low-certainty evidence). The highest ranked surgical method was suction aspiration after cervical preparation. The highest ranked non-surgical treatment was mifepristone plus misoprostol. All surgical methods were ranked higher than medical methods, which in turn ranked above expectant management. Since there is no evidence that any approach results in different long-term outcomes, discussing all clinically appropriate and locally available options with patients represents an important step in EPL management. According to the ACOG Bulletin, in the absence of medical complications or symptoms requiring urgent surgical evacuation, treatment plans can safely accommodate patient treatment preferences [1].

To date, when counseling patients about the most personalized treatment option, a crucial question remains the rate of response to medical treatment and, more importantly, whether there are specific factors able to predict a good response to misoprostol. To address this, our study aimed to determine whether clinical characteristics of women with EPL could be associated with a successful response to medical treatment.

## 2. Methods

This observational prospective study was conducted at the Early Pregnancy Loss Unit of the Department of Obstetrics and Gynecology, Fondazione A. Gemelli IRCCS Rome, Italy. All consecutive women aged ≥18 years with a diagnosis of EPL, defined as anembryonic gestation or embryonic/fetal spontaneous demise between 5 and 12 weeks of gestation, were included. Gestational age was determined by ultrasound measurement of the crown rump length (CRL) of the embryo or fetus [20]. Amenorrhea age was calculated from the first day of the last menstrual period.

The protocol was approved by the Institutional Reviewer Board (Prot. N. 3868), and informed consent was obtained from all patients. Data collection and management were conducted using REDCap (Research Electronic Data Capture) version 14.0.10, a secure, web-based software platform hosted at https://redcap-irccs.policlinicogemelli.it/ (accessed on 1 March 2022) [21,22]. Only registered study investigators or data managers were granted login credentials to access the REDCap web platform for data entry and management.

Women diagnosed with EPL attended our EPL clinic for an initial obstetrical consultation and pelvic ultrasound. The following exclusion criteria were applied: clear evidence of complete abortion, defined as the passage of all missed miscarriage; inevitable abortion, characterized by symptoms of threatened pregnancy loss (e.g., bleeding and cramping), and an open cervix at the initial obstetric ultrasound evaluation; clinical or ultrasonographic conditions requiring D&C as first-line treatment, including sepsis, heavy bleeding, or hemodynamic instability; ultrasound suspicion of gestational trophoblastic disease, identified as an enlarged uterus filled with a heterogeneous echogenic mass with multiple hypoechoic foci, small cystic spaces, and hydropic chorionic villi, forming the typical “cluster of grapes” appearance [23,24]; or history of adverse reaction or medical contraindications to misoprostol, including coagulopathies, anticoagulant therapy, or hemoglobin values ≤ 10 g/dL. After excluding the above conditions, and in the absence of medical complications or symptoms requiring urgent surgical evacuation, patients were offered specific counselling about all available EPL management options (expectant, D&C or medical management), allowing for personalized treatment decisions based on individual, family, and psychological factors. Expectant management consisted of a 7-day observation period, until the spontaneous passage of missed miscarriage. Surgical management was based on planned D&C. Medical treatment included an initial vaginal administration of 800 mcg of misoprostol, and a follow-up visit with ultrasound evaluation (after 3–5 days). The success of medical treatment was defined as the complete expulsion of spontaneous miscarried tissue after one or two doses of misoprostol. Follow-up evaluation included transvaginal ultrasound to assess endometrial thickness: an endometrial thickness < 20 mm was considered indicative of an adequate response to medical treatment, with no need for further intervention; conversely, an endometrial thickness ≥ 20 mm meant an inadequate response, and a second dose of vaginal misoprostol was administered, followed by another follow-up visit with ultrasound (after 3–5 days). At the subsequent assessment, an endometrial thickness ≤ 15 mm indicated a successful response to medical treatment, with no need for further intervention. If the endometrial thickness remained > 15 mm, the response was considered inadequate, and the patient was then offered a specific counselling to decide the most appropriate management option between surgery and active surveillance. To establish the role of obstetrical, laboratory and ultrasonographic variables, the following parameters were recorded: type of conception (spontaneous, assisted reproductive technology), amenorrhea and gestational age at miscarriage; white blood cell, neutrophils, lymphocytes and platelets count, mean platelet volume; gestational sac mean diameter, CRL measurement. After classifying patients into one of the three EPL management groups (A: expectant management; B: D&C; C: medical treatment), we evaluated whether any clinical, laboratory or ultrasound features could predict the response to medical treatment with misoprostol.

Sample size was calculated according to the population proportion formula with a finite population correction [25]. Assuming a total annual population of approximately 4000 pregnant women referred to our institution, an estimated 20% incidence of EPL [1,2,3,4], a 90% confidence level, and a 5% margin of error, the minimum required sample size was 167 patients.

Patient characteristics were described using appropriate descriptive statistics, with normality assessed using the Shapiro–Francia test. Patients were grouped according to the type of treatment: expectant management (group A), elective D&C (group B), and medical treatment (group C). A separate analysis was performed within the medical treatment group to distinguish successful from unsuccessful cases. The success rate of medical treatment was calculated with a 95% Confidence Interval (CI).

Comparisons were performed using the Kruskal–Wallis test for group comparisons (A versus B versus C); the Mann–Whitney U test for successful versus unsuccessful misoprostol treatment (continuous variables); and Pearson’s χ^2^ test and Fisher’s exact test for categorical variables. For group C, a multivariable analysis was planned for variables with *p* < 0.05 in the univariable analysis. Receiver Operating Characteristic (ROC) analysis was performed to evaluate the ability of continuous obstetrics parameters to predict treatment success, as well as to determine the optimal cut-off value to differentiate responders from non-responders. The best cut-off value was determined using Youden’s method [26]. Sensitivity, specificity, accuracy, positive predictive value (PPV) and negative predictive value (NPV) were also calculated, all presented with two-sided 95% CIs.

Statistical analysis was performed using STATA software (STATA/BE 17.0 for Windows, Stata Corp LP, College Station, TX, USA). Two-sided tests were used, with *p* < 0.05 considered significant, except in case of multiple comparisons, where Bonferroni’s correction was applied. No imputation was carried out for missing data.

We performed a post hoc power analysis to evaluate the adequacy of our sample size for detecting a meaningful difference in medical therapy outcome (success versus unsuccess). Using a one-sided Mann–Whitney U (Wilcoxon Rank-Sum) test, assuming a normal distribution of the data, a significance level (alpha) of 0.05, and known standard deviations (SD) in both groups, we calculated the statistical power based on the observed group sizes of 54 and 10. The mean difference in amenorrhea age between the groups was 60.7 days versus 66.8 days, yielding an effect size of −6.1 days with SD of 10 days and 7.3 days, respectively. Under these conditions, the estimated power was 74%. While this falls slightly below the conventional threshold of 80%, it still indicates a moderate ability to detect the observed effect. Power calculation was performed with NCSS Statistical Software ((2021) PASS (Version 2021.0.6)).

## 3. Results

**Figure 1:**
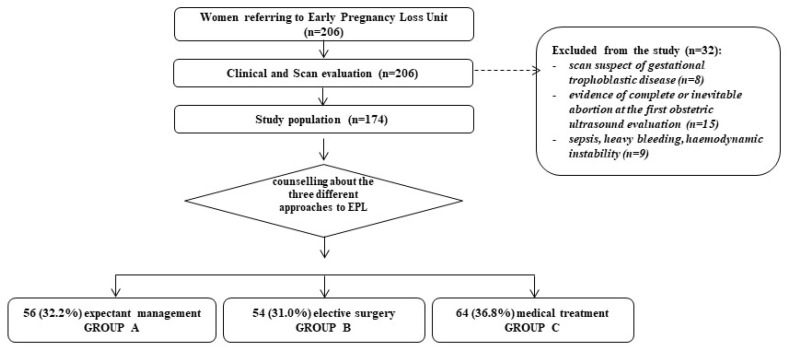
Representative flowchart of the women included in the prospective observational analysis. EPL: Early pregnancy loss.

From March 2022 to December 2022, 206 patients agreed to participate in the study (Figure 1). However, 32 women were excluded due to several criteria: evidence of complete or inevitable abortion at the first obstetric ultrasound evaluation and specific conditions requiring D&C (sepsis, heavy bleeding, hemodynamic instability, or suspicion of gestational trophoblastic disease). Among the remaining 174 patients, 56 (32.2%) underwent expectant management, 54 (31.0%) underwent elective D&C, and 64 (36.8%) received medical treatment. The clinical characteristics and obstetric history of the included women are reported in Table 1. The distribution of women across the three treatment groups (A: expectant management; B: dilatation and curettage; C: medical treatment) was homogeneous in terms of previous pregnancy, type of delivery, and previous gynecological surgery. However, women undergoing medical treatment were significantly younger compared with those in the other two groups [median (min–max), years: 38 (16–47) versus 37 (19–45) versus 35 (20–45) for groups A, B, and C, respectively; *p* = 0.026]. Additionally, the number of outpatient visits was significantly higher in the medical treatment group [median (min–max): 1 (0–2) vs. 1 (0–2) vs. 3 (1–5) for groups A, B, and C, respectively; *p* = 0.0001].

The obstetrical characteristics of the entire study population are described in Table 2. The amenorrhea and gestational ages at miscarriage were significantly lower in the medical treatment group [median (min–max), days: 66 (28–90) vs. 70 (41–117) vs. 63 (35–84) for groups A, B, and C, respectively; *p* = 0.004] and [median (min–max), days: 49 (28–80) vs. 55 (35–93) vs. 49 (35–69) for groups A, B, and C, respectively; *p* = 0.004]. Similarly, CRL measurement was lower in the medical treatment group [mean (min–max), mm: 8.4 (2.3–23) vs. 13 (2.2–75) vs. 5 (2.2–33) for groups A, B and C, respectively; *p* = 0.004]. A diagnosis of anembryonic gestation (blighted ovum) was more frequently observed in the expectant management group compared to the surgical or medical treatment groups (46.4% vs. 74.1% and 73.4% for groups B and C, respectively; *p* = 0.002).

**Figure 2:**
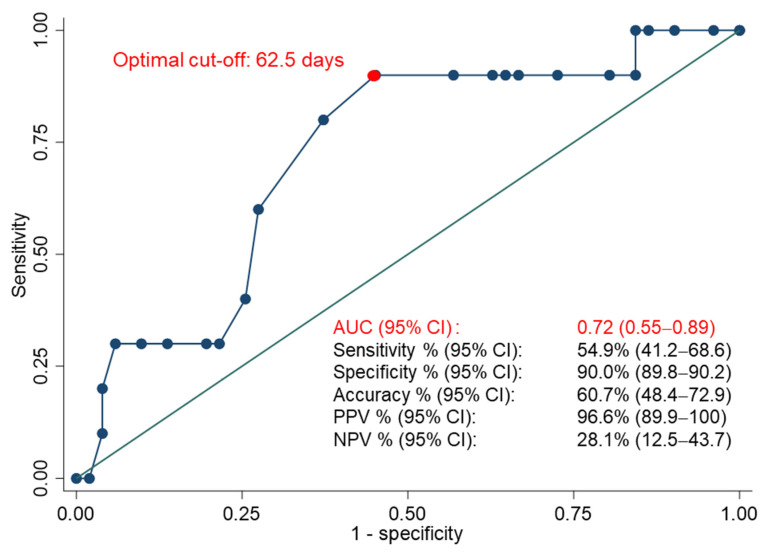
Receiver Operating Characteristic (ROC) curve of amenorrhea age in predicting the success of medical treatment. Performances metrics are described in terms of sensitivity, specificity, positive predictive value (PPV) and negative predictive value (NPV). The area under the curve (AUC) and performance metrics are presented with Confidence Intervals (CIs). The dot represents the optimal cut-off according to Youden’s method, which maximizes the sum of sensitivity and specificity [26].

Among the 64 women who underwent medical treatment, the overall response rate was 84.4% (54/64) (95% CI: 75.5–93.3%). No statistically significant differences were found between responsive and unresponsive patients in terms of age, previous pregnancies, previous type of delivery (vaginal or cesarean section), number of prior terminations of pregnancy, previous miscarriages, prior D&C, previous gynecological surgery (myomectomy and/or salpingectomy), or the number of outpatient visits (Table 3). When analyzing the obstetrical, laboratory and ultrasound characteristics of women with EPL receiving medical treatment, the only parameter found to be significantly associated with a successful response to treatment was amenorrhea age (Table 4). Responsive patients had a median amenorrhea age of 62 days (min–max:35–84) versus 65 days (min–max: 53–77) in unresponsive ones (*p* = 0.027). According to ROC analysis, the best cut-off for predicting a successful response to medical treatment was 62.5 days, with an AUC (95% CI) of 0.72 (0.55–0.89). An amenorrhea age ≤ 62.5 days was associated with a successful response, with a specificity (95% CI) of 90.0% (89.8–90.2) and a sensitivity (95% CI) of 54.9% (41.2–68.6) (Figure 2). Due to the absence of other statistically significant predictors of treatment success, the planned multivariable analysis was not performed. Among women undergoing medical treatment, 75.0% (48/64) received a single vaginal administration of misoprostol, with a response rate of 93.8% (45/48). In contrast, 25.0% (16/64) required a second dose of misoprostol, administered 3–5 days after the first dose. In these cases, the response rate was significantly lower at 56.2% (9/16; Table 5). Following medical treatment, 6.3% of patients required emergency room evaluation for complications, though none required hospitalization, with no statistically significant differences between responsive and unresponsive patients (5.6% vs. 10.0%, respectively; *p* = 0.594; Table 5). The overall hospital admission rate for complications (e.g., hemorrhage, pelvic pain) among medically treated patients was 9.4%. However, a significant difference was noted between responders and non-responders: only 1.9% (1/54) of responders required hospitalization (due to hemorrhage requiring blood transfusion), compared to 50.0% (5/10) of non-responders (*p* < 0.0001). The median hospital stay for non-responders was one day (Table 5).

## 4. Discussion

In the present study we considered women with EPL and evaluated whether, after medical treatment with vaginal misoprostol, specific clinical or hematological features could be associated with a successful response. Women with EPL received counselling about possible treatment options and were homogeneously distributed across the three treatment subgroups (expectant, surgical, and medical management). No significant differences were found in terms of previous pregnancies, type of delivery, or previous gynecological surgeries among the groups. Patients in the medical treatment group were significantly younger and required a higher number of outpatient visits compared with the other two groups. This observation might require further investigation with studies focusing on the reason leading to the choice of the EPL management. Additionally, we might suggest that repeated follow-up visits might provide longer supportive care, potentially helping those women needing long-term psychological support. We found a success rate of 84.4% (95% CI: 75.5–93.3) for the misoprostol treatment. These results are consistent with previously reported findings [12,27]. Zhang et al. [12] reported a misoprostol success rate of 84 percent (95% CI: 81–87%) by day 30. They reported a complete expulsion of products of conception after one dose in 71% of the women. Among those who received a second dose, the success rate was 60%. Accordingly, our findings showed that 75% of treated women required only a single vaginal administration of misoprostol, while the remaining 25% needed a second dose 3–5 days later. In these latter cases the response rate decreased significantly to 56.2%. Of note, different trials have demonstrated that pre-treatment with mifepristone is more effective than misoprostol alone in the management of missed miscarriage in the absence of substantial differences in adverse effects, clinical complications, or treatment acceptability between mifepristone plus misoprostol versus misoprostol alone [28,29,30,31]. Nevertheless, at the time of the research, in our hospital we used misoprostol alone given its advantages including low cost, long shelf life, no need for refrigeration, no need for patients to a repeated outpatient visit two days apart to complete the first treatment cycle. Also, high access restrictions to mifepristone in our center limited the use of mifepristone. In contrast to Zhang et al., which found no significant association between gestational age at the time of treatment and success rate (*p* = 0.67 for the misoprostol group) [12], we have found that medical treatment success was significantly associated with an earlier amenorrhea age. Specifically, responders had a median amenorrhea age of 62 days (min–max: 35–84), compared to 65 days (min–max: 53–77) in non-responders (*p* = 0.027). ROC analysis identified an optimal cut-off of 62.5 days, with an AUC (95% CI) of 0.72 (0.55–0.89). An amenorrhea age ≤ 62.5 days predicted a successful response to medical treatment with a specificity of 90.0% (95% CI: 89.8–90.2) and a sensitivity of 54.9% (95% CI: 41.2–68.6). The absence of statistically significant differences between responsive and unresponsive patients in terms of age, previous pregnancies, previous type of delivery, number of prior terminations of pregnancy, previous miscarriages, prior D&C, previous gynecological surgery, or the number of outpatient visits, suggests that these clinical features do not have a substantial impact on treatment response. However, these results must be confirmed in larger studies. Our research also suggests that laboratory values of immune activation are unlikely to serve as useful predictors of misoprostol treatment. Sonalkar et al. identified as possible predictors of successful response to misoprostol the vaginal bleeding within the past 24 h and a parity > 1, even with a low predictive value [29].

Additionally, the single-center design may limit the generalizability of our results to broader populations. Future studies with larger, more diverse cohorts are needed to validate these findings and strengthen the predictive model.

Our study has several limitations that warrant consideration. Most notably, the sample size for medical therapy group was relatively small, particularly in the subgroup experiencing treatment failure, which comprised only 10 cases. This low event rate limits the statistical power of our predictor analysis and increases the risk of Type II error, potentially obscuring associations that may exist. Although a post hoc power calculation indicated a moderate power of 74% to detect the observed effect size, this remains below the conventional 80% threshold and should be interpreted with caution. Consequently, our finding that amenorrhea age is the sole predictor of treatment failure should be considered exploratory rather than definitive. The limited sample size may also have contributed to the moderate discriminative ability of the ROC curve for amenorrhea age, with an AUC of 0.72 [32]. Nevertheless, our sample size was designed to reflect a representative population of women experiencing EPL, and we believe it is valuable to report our findings as a contribution to the ongoing effort to identify predictors of successful medical management. In this context, we also explored hematological markers of immune activation, although these did not emerge as significant predictors in our cohort.

Another limitation is the potential for selection bias, as treatment plans were determined in part by patient preference. While this approach aligns with patient-centered care, it may have introduced confounding factors related to individual motivations or expectations. Furthermore, our results do not allow us to distinguish whether the treatment choice was primarily influenced by patient preferences, counselling or clinical recommendation. This might limit the interpretability of these findings even if it was beyond the aim of our study.

Additionally, we excluded patients presenting with heavy bleeding or sepsis to ensure a homogeneous study population and to isolate the effect of the intervention. While necessary for methodological consistency, this exclusion may limit the applicability of our findings to more complex clinical scenarios.

Importantly, we did not control for psychological variables, which are known to influence both treatment outcomes and patient decision-making. Factors such as emotional distress, grief, and mental health status may significantly affect compliance and perceived success [33,34]. Future studies should incorporate validated psychological assessment tools—such as the Revised Impact of Miscarriage Scale, the Perinatal Grief Scale, and the Montgomery–Åsberg Depression Rating Scale—to better understand the interplay between emotional wellbeing and treatment response.

Finally, the single-center design may limit the generalizability of our results to broader populations. Larger, multicenter studies with more diverse populations are needed to validate our results and to refine predictive models for individualized care in early pregnancy loss.

In conclusion, optimal management of EPL should consider different factors, including clinical characteristics, psychological wellbeing, and patient preferences. In our study, the overall success rate of medical treatment with misoprostol was 84.4% (95% CI: 75.5–93.3), and a significantly earlier amenorrhea age was associated with treatment success. An amenorrhea age ≤ 62.5 days may represent a useful predictor, with a specificity of 90.0%, although its moderate sensitivity warrants cautious interpretation. Future research should explore the predictive role of additional variables, including psychological factors, to improve personalized treatment approaches for EPL.

## Figures and Tables

**Table 1:** Clinical characteristics and obstetric history of the study population across the three EPL treatment groups.

Characteristics	Group A Expectant Management	Group B Elective Surgery	Group C Medical Treatment	*p*-Value
Number of patients	56	54	64	
Age, years				**0.026**
Median (min–max)	38 (18–47)	37 (19–45)	35 (20–45)	
Mean ± SD	37.3 ± 5.9	36 ± 6.1	34.8 ± 4.8	
Number of previous pregnancies				
Median (min–max)	2 (1–7)	2 (0–7)	2 (0–7)	0.902
0	0 (0)	1 (1.9)	2 (3.1)	0.181
1	12 (21.4)	17 (31.5)	20 (31.3)	
2	25 (44.6)	18 (33.3)	14 (21.9)	
>2	19 (33.9)	18 (33.3)	28 (43.8)	
Vaginal delivery				0.078
0	30 (53.6)	42 (77.8)	45 (70.3)	
1	21 (37.5)	11 (20.4)	16 (25.0)	
2	5 (8.9)	1 (1.9)	3 (4.7)	
Cesarean section				0.737
0	46 (82.1)	44 (81.5)	55 (85.9)	
1	9 (16.1)	8 (14.8)	6 (9.4)	
2	1 (1.8)	2 (3.7)	3 (4.7)	
Termination of pregnancy				0.502
0	55 (98.2)	50 (92.6)	58 (90.6)	
1	1 (1.8)	3 (5.6)	4 (6.3)	
2	0 (0)	1 (1.9)	2 (3.1)	
Number of miscarriages †				
Median (min–max)	1 (1–5)	1 (1–5)	1 (1–6)	0.414
1	36 (64.3)	28 (51.9)	37 (57.8)	0.507
2	13 (23.2)	16 (29.6)	13 (20.3)	
>2	7 (12.5)	10 (18.5)	14 (21.9)	
Previous dilation & curettage				0.196
0	47 (83.9)	35 (64.8)	46 (71.9)	
1	7 (12.5)	12 (22.2)	13 (20.3)	
≥2	2 (3.6)	7 (13)	5 (7.8)	
Myomectomy	3 (5.4)	1 (1.9)	1 (1.6)	0.400
Salpingectomy	3 (5.4)	0 (0)	1 (1.6)	0.153
Number of outpatients visits	1 (0–2)	1 (0–2)	3 (1–5)	**0.0001**

Results are presented as n (%), median (min–max) as appropriate, except where indicated. *p*-values were evaluated according to Pearson’s Chi-square test or Kruskal–Wallis test as appropriate. Bold font highlights statistically significant value: *p* < 0.05 or *p* < 0.05/number of comparisons in case of characteristics requiring Bonferroni’s correction. SD: Standard Deviation. † Including the current one.

**Table 2:** Obstetrical, laboratory and ultrasonographic characteristics of the study population according to the miscarriage management approach.

Characteristics	Group A Expectant Management	Group B Elective Surgery	Group C Medical Treatment	*p*-Value
Number of patients	56	54	64	
Conception				0.856
Spontaneous	51 (91.1)	50 (92.6)	60 (93.8)	
Assisted reproductive technology	5 (8.9)	4 (7.4)	4 (6.3)	
Amenorrhea age at miscarriage, days †				**0.004**
Median (min–max)	66 (28–90)	70 (41–117)	63 (35–84)	
Mean ± SD	65.9 (13.5)	70.7 (14.3)	61.7 (9.8)	
Gestational age at miscarriage, days ‡				**0.025**
Median (min–max)	49 (28–80)	55 (35–93)	49 (35–69)	
Mean ± SD	50.2 (11.1)	55.6 (12.2)	49 (8.5)	
White Blood Cells count (×10^9^/L) (Mean + SD)	7.99 ± 1.98	8.35 ± 3.12	8.39 ± 3.12	0.702
Neutrophils (×10^9^/L)(Mean + SD)	5.98 ± 1.75	6.01 ± 1.98	5.45± 1.76	0.169
Lymphocytes, ×10^9^/L (Mean + SD)	1.98± 1.1	1.85± 0.8	2.03 ± 1.5	0.706
Platelets (×10^9^/L) (Mean ± SD)	251.81 ± 34.50	255.78 ± 38.51	253.68 ± 57.50	0.887
Mean Platelet Volume, fl, (Mean ± SD)	8.4 ± 1.2	8.1 ± 1.5	7.9 ± 1.5	0.15
Anembrionic gestation (blighted ovum)	26 (46.4)	40 (74.1)	47 (73.4)	**0.002**
Morphometric evaluation:				
Gestational sac mean diameter, mm ¶	19 (9–50)	28.2 (8–53)	23 (9–81.5)	0.086
Crown Rump Length (mean), mm	8.4 (2.3–23)	13 (2.2–75)	5 (2.2–33)	**0.004**

Results are presented as n (%), median (min–max) as appropriate, except where indicated. *p*-values were evaluated according to Pearson’s Chi-square test or Kruskal–Wallis test as appropriate. Bold font highlights statistically significant value: *p* < 0.05 or *p* < 0.05/number of comparisons in case of characteristics requiring Bonferroni’s correction. SD: Standard Deviation. † Information available for 169/174 patients. ‡ Information available for 108/174 patients. ¶ Information available for 85/174 patients.

**Table 3:** Clinical characteristics and obstetric history of patients undergoing medical therapy according to treatment success or failure.

Characteristics	Responsive Group	Unresponsive Group	*p*-Value
Number of patients	54	10	
Age, years			0.623
Median (min–max)	34.5 (20–45)	37 (27–41)	
Mean ± SD	34.7 ± 4.9	35.4 ± 4.4	
Number of previous pregnancies			
Median (min–max)	2 (0–7)	2 (1–7)	0.872
0	2 (3.7)	0 (0)	0.856
1	17	3	
2	11	3	
>2	24	4	
Vaginal delivery			0.321
0	36	9	
1	15	1	
2	3	0	
Cesarean section			0.288
0	48	7	
1	4	2	
2	2	1	
Termination of pregnancy			0.322
0	50	8	
1	3	1	
2	1	1	
Number of miscarriages †			
Median (min–max)	1 (1–6)	1 (1–5)	0.605
1	30	7	0.624
2	12	1	
>2	12	2	
Previous D & C			0.653
0	40	6	
1	10	3	
≥2	4	1	
Myomectomy	1	0	0.664
Salpingectomy	1	0	0.664
Number of outpatients visits	3	3	0.614

Results are presented as n (%), median (min–max) as appropriate, except where indicated. *p*-values were evaluated according to Pearson’s Chi-square test or Mann–Whitney U test as appropriate. The statistical level of significance was set at *p* < 0.05 or *p* < 0.05/number of comparisons in case of characteristics requiring Bonferroni’s correction. SD: Standard Deviation. † Including the current one.

**Table 4:** Obstetrical, laboratory, ultrasound characteristics and hospital outcomes according to treatment response in women undergoing medical approach.

Characteristics	Responsive Group	Unresponsive Group	*p*-Value
Number of patients	54	10	
Conception			0.374
Spontaneous	50	10	
Assisted reproductive technology	4	0	
Amenorrhea age at miscarriage, days ‡			**0.027**
Median (min–max)	62 (35–84)	65 (53–77)	
Mean ± SD	60.7 ± 10	66.8 ± 7.3	
Gestational age at miscarriage, days §			0.961
Median (min–max)	49 (35–69)	49 (41–56)	
Mean ± SD	48.9 ± 8.9	49.1 ± 6.1	
White Blood Cells count (×10^9^/L) (Mean + SD)	8.12 ± 2.56	8.45 ± 2.56	0.709
Neutrophils (×10^9^/L) (Mean + SD)	5.98 ± 2.12	5.87± 2.12	0.88
Lymphocytes, ×10^9^/L (Mean + SD)	1.89 ± 1.85	2.45 ± 2.12	0.450
Platelets (×10^9^/L) (Mean ± SD)	249.79 ± 61.50	248.78 ± 45.6	0.952
Mean Platelet Volume, fl (Mean ± SD)	8.1 ± 1.9	8.5 ± 1.5	0.471
Anembrionic gestation (blighted ovum)	39	8	0.609
Morphometric evaluation			
Gestational sac mean diameter, mm ¶	22.8 (9–81.5)	29.5 (12.3–40.6)	0.379
Crown rump length mean diameter, mm §	5 (2.2–33)	9.6 (4.3–18)	0.182

Results are presented as n (%), median (min–max) as appropriate, except where indicated. *p* values were evaluated according to Pearson’s Chi-square test or Mann–Whitney U test as appropriate. Bold font highlights statistically significant value: *p* < 0.05 or *p* < 0.05/number of comparisons in case of characteristics requiring Bonferroni’s correction. SD: Standard Deviation. ER: Emergency Room. ‡ Information available for 61/64 patients. § Information available for 44/64 patients. ¶ Information available for 39/64 patients.

**Table 5:** Hospital outcomes according to treatment response in women undergoing medical approach.

Characteristics	Responsive Group	Unresponsive Group	*p*-Value
Number of medical therapies administered			**<0.0001**
1	45	3	
2	9	7	
Post therapy hospital outcomes			
Hospital stay, days			**<0.0001**
Median (min–max)	0 (0–1)	1 (0–1)	
ER after medical therapy for complications without readmission	3 (5.6)	1 (10.0)	0.594
Readmission after medical therapy for complications	1 (1.9)	5 (50.0)	**<0.0001**
Dilation & curettage after medical therapy	0 (0)	8 (80.0)	**<0.0001**

Results are presented as n (%), median (min–max) as appropriate, except where indicated. *p*–values were evaluated according to Pearson’s Chi-square test or Mann–Whitney U test as appropriate. Bold font highlights statistically significant value: *p* < 0.05 or *p* < 0.05/number of comparisons in case of characteristics requiring Bonferroni’s correction. ER: Emergency Room.

## Data Availability

The data supporting the findings of this study are available from the corresponding author upon reasonable request.
